# Cytochrome c Oxidase Subunit COX4-1 Reprograms Erastin-Induced Cell Death from Ferroptosis to Apoptosis: A Transmitochondrial Study

**DOI:** 10.3390/antiox15010040

**Published:** 2025-12-28

**Authors:** Claudia R. Oliva, Susanne Flor, Corinne E. Griguer

**Affiliations:** Free Radical & Radiation Biology Program, Department of Radiation Oncology, University of Iowa, Iowa City, IA 52242, USA; claudia-oliva@uiowa.edu (C.R.O.); susanne-flor@uiowa.edu (S.F.)

**Keywords:** ferroptosis, apoptosis, COX4-1 isoform, transmitochondrial cybrids, erastin, glioma

## Abstract

Ferroptosis is an iron-dependent, oxidative form of regulated cell death that has emerged as a therapeutic vulnerability in glioblastoma; however, the mitochondrial determinants that govern ferroptotic sensitivity remain poorly defined. Cytochrome c oxidase (CcO/Complex IV), a key regulator of mitochondrial respiration, contains two isoforms of subunit IV (COX4): COX4-1, a housekeeping isoform, and COX4-2, a stress-inducible variant. We previously found that COX4-1 expression protects glioma cells from erastin-induced ferroptosis, suggesting that mitochondria influence cell-death decisions independently of canonical ferroptotic regulators. Here, we used CRISPR-generated POLG-knockout ρ^0^ cells and transmitochondrial cybrids to isolate mitochondrial from nuclear contributions to ferroptosis sensitivity. Cybrids reconstituted with COX4-1-containing mitochondria restored CcO activity and recapitulated the ferroptosis-resistant phenotype, whereas COX4-2 cybrids remained insensitive to erastin. COX4-1 cybrids exhibited reduced labile iron, diminished cystine uptake, and low expression of SLC7A11 and GPX4, yet underwent apoptosis rather than ferroptosis upon erastin treatment. These findings demonstrate that mitochondrial COX4-1 rewires redox metabolism and diverts cell-death signaling away from ferroptosis toward apoptosis. Our results identify isoform-specific mitochondrial composition as a previously unrecognized determinant of regulated cell death and highlight COX4-1-driven mitochondrial remodeling as a potential mechanism of therapeutic resistance in glioblastoma.

## 1. Introduction

Ferroptosis, a regulated form of cell death that is mechanistically distinct from apoptosis and necrosis, is characterized by iron-dependent oxidative stress and lipid peroxidation that irreversibly damages cell membranes [1,2,3]. This cell death pathway has been implicated in cancer biology, and targeting ferroptosis has gained particular attention as a strategy for enhancing cancer therapy and overcoming the adaptive therapy resistance that severely restricts treatment efficacy in aggressive cancers, including glioblastoma (GBM). However, the complex cell type- and environment-specific molecular pathways involved in regulating ferroptosis remain to be fully elucidated [4,5,6,7].

Although myriad pathways contribute to the ferroptotic network, ferroptosis is primarily driven by the interaction between the unbound Fe^2+^ of the intracellular labile iron pool (LIP) and hydrogen peroxide, which produces the lipid reactive oxygen species (ROS) that mediate lipid peroxidation. Under homeostatic conditions, the antioxidant glutathione (GSH) controls the accumulation of lipid ROS, and thus the potential for membrane damage, by reducing glutathione peroxidase 4 (GPX4). In its reduced state, GPX4 converts the lipid ROS to non-toxic lipid alcohols. GSH is a product of cystine metabolism; therefore, the GSH/GPX4-mediated control of ferroptosis is in turn regulated by the cellular uptake of cystine by SLC7A11, a subunit of the cystine/glutamate antiporter known as system X_c_^−^. The small molecule erastin is a prototypical ferroptosis inducer that promotes this process by blocking cystine uptake through system X_c_^−^, thereby leading to the unchecked accumulation of lipid ROS. For this reason, erastin has gained much attention as a potential therapeutic adjuvant in the treatment of cancer [1,2,8,9].

Mitochondrial bioenergetics, redox balance, and superoxide (O_2_^•−^) generation have also been proposed as regulators of ferroptotic sensitivity [10,11,12], yet the precise mitochondrial determinants that influence ferroptosis remain poorly understood [11,13,14,15]. In particular, the remodeling of electron transport chain (ETC) complexes and their isoform-specific regulation may play a central role in shaping cellular fate under oxidative stress. Cytochrome c oxidase (CcO, Complex IV), the terminal enzyme of the mitochondrial respiratory chain, exists with alternative isoforms of subunit IV (COX4): COX4-1, a ubiquitous housekeeping isoform necessary for coupling electron transport to the production of ATP, and COX4-2, an inducible form associated with hypoxic or stress conditions [16,17,18]. We recently demonstrated that expression of the COX4-1 isoform increases mitochondrial respiration and confers resistance to erastin-induced ferroptosis in glioma cells, even in the absence of canonical ferroptotic machinery associated with ferroptosis resistance [19]. These findings suggest that the mitochondrial COX4-1 isoform rewires cellular death decisions, diverting stress responses away from ferroptosis.

To dissect the mitochondrial contribution to this phenotype, we employed transmitochondrial cybrids to isolate mitochondrial from nuclear effects. Here, we demonstrate that mitochondria enriched in COX4-1 are sufficient to reproduce the ferroptosis-resistant phenotype, providing direct evidence that isoform-specific mitochondrial composition governs ferroptosis sensitivity. Importantly, these results establish mitochondrial isoform switching as a previously unrecognized determinant of cell death fate, with broad implications for therapeutic resistance and metabolic adaptation in disease.

## 2. Materials and Methods

### 2.1. Generation of Mitochondria-Depleted Cells and Transmitochondrial Cybrids

Human glioma cell line U251 was originally obtained from Dr. G. Yancey Gillespie (University of Alabama at Birmingham, Birmingham, AL, USA) and authenticated using the short tandem repeat (ATCC, STR service, Manassas, VA, USA). Cells were grown as we previously described in DMEM F-12 medium plus L-glutamine supplemented with 7% heat-inactivated FBS [20,21]. Cells were incubated at 37 °C in a humidified atmosphere containing 5% CO_2_. The generation of transgenic U251 cells overexpressing only COX4-1 or COX4-2 was previously described [20,22].

To create mitochondrial-deficient cells (ρ^0^ cells), the gene encoding the catalytic subunit of DNA polymerase δ (POLG) was knocked out in glioma cells using the Edit-R Human POLG mCMV All-in-One lentiviral sgRNA set (three-sgRNA pool; catalog #VSGH11940-15EG5428, Horizon/Dharmacon, Lafayette, CO, USA) delivered as prepackaged lentiviral particles. After transduction, cells were maintained in medium supplemented with 50 µg/mL uridine and 1 mM sodium pyruvate. After 24 h, the medium was replaced; 48–72 h post-transduction selection was initiated using puromycin (0.5 µg/mL) for 5–7 days to enrich for transduced cells. For clonal knockout (KO) lines, puromycin-resistant cells were diluted to single cells and expanded.

To obtain transmitochondrial cybrids, transgenic U251 cells were enucleated as previously described [21,23,24,25]. Briefly, cells were treated with cytochalasin B (10 μg/mL) and then layered on an isopycnic Percoll gradient. The tubes were then centrifuged at 19,000 rpm for 70 min in a Sorvall SS-34 fixed-angle rotor in a RC-5B centrifuge maintained at 30 °C. The cytoplasts were then fused with ρ^0^ cells by adding a solution of 50% polyethylene glycol (MW 1450, Sigma-Aldrich, St. Louis, MO, USA, catalog #P-5402) in phosphate buffer. Two weeks after fusion, cybrids were plated at low density for clonogenic selection.

### 2.2. PCR Analysis

Total cellular DNA was extracted using the QIAamp DNA Mini Kit (QIAGEN, Germantown, MD, USA). Amplification of mitochondrial DNA (mtDNA) was performed with the human mtDNA-specific primers COXI (NC_011137.1): forward 5′-TGGAGCCTCCGTAGACCTAA-3′ and reverse 5′-TCCGAGCCTGGTAGGATAA-3′. As a control, the nuclear gene actin (ACTB, NC_000007.14) was amplified using the following primers: forward 5′-CATGTACGTTGCTATCCAGGC-3′ and reverse 5′-CTCCTTAATGTCACGCACGAT-3′. Each PCR reaction contained 100 ng of template DNA, 5 µM of each primer, and Platinum Taq DNA Polymerase (Invitrogen, Carlsbad, CA, USA). The cycling conditions were as follows: initial denaturation at 94 °C for 2 min, followed by 40 cycles of 94 °C for 1 min (denaturation), 55 °C for 1 min (annealing), and 72 °C for 1 min (extension), with a final extension at 72 °C for 10 min. PCR products were analyzed on 1% agarose gels.

### 2.3. SDS-PAGE and Immunoblotting

SDS-PAGE and immunoblotting were performed as previously described [20,22]. Briefly, mitochondrial extracts or total lysates were collected and loaded and electrophoresed in 4–20% TGX mini precast polyacrylamide gels (Bio-Rad, Hercules, CA, USA). Proteins were then transferred to polyvinyl fluoride membranes, and the membranes were incubated with the indicated antibodies. Membranes were developed with clarity enhanced chemiluminescent (ECL) HRP substrates (Bio-Rad) and exposed to Autoradiography Classical X-Ray Film (Research Products Internationals, Mount Prospect, IL, USA). The following primary antibodies were used: anti-COX4-1 (1:1000; ab14744, Abcam, Waltham, MA, USA), anti-COX4-2, anti-POLG, anti-citrate synthase, anti-SLC7A11/xCT, anti-GPX4, and anti-ACTB (1:1000, 11463-AP and 16131-AP; 1:5000, 83670-1-RR; 1:2000, 26864-1-AP; 1:5000, 67763-1-Ig; and 1:10,000, 66009-1-Ig; Proteintech Group, Rosemont, IL, USA).

### 2.4. Mitochondrial Preparation and CcO Activity

Mitochondrial fractions were prepared from cultured cells as we previously described [20,26]. Briefly, cells were resuspended in ice-cold PBS containing 0.86 mg/mL of digitonin and incubated on ice for 10 min to solubilize the plasma membrane. After incubation, ice-cold PBS was added to dilute the digitonin 50-fold, and the solution was centrifuged at 17,000× *g* for 7 min. Pellets were then washed once with ice-cold PBS and centrifuged again. Cell pellets were stored at −80 °C until use. CcO activity was determined, with results normalized to citrate synthase activity, as previously described [27,28].

### 2.5. GSH Determination

GSH was determined as previously described [29]. Briefly, cells were seeded in 96-well clear-bottom microplates at a subconfluent density and allowed to adhere overnight. Cells were then treated with erastin (0, 2.5, and 5 µM for 24 h) or with buthionine sulfoximine (BSO) or menadione (MD) to deplete GSH. After treatment, cells were washed once with Hank’s Balanced Salt Solution (HBSS, Thermo Fisher Scientific, Waltham, MA, USA) at room temperature. Monochlorobimane (MCB, 2 µM–20 µM) was added to each well and fluorescence was measured every 10 min (up to 60 min) with a Tecan Infinite 200 microplate spectrofluorometer (Morrisville, NC, USA) at ~380 nm (excitation) and ~461 nm (emission). Background (wells without cells or without MCB) was subtracted. In experiments assessing modulators (BSO, MD, or erastin), the relative change in fluorescence compared with that in the untreated control wells was used to determine the change in GSH content. Data were normalized to the mean cell survival fraction following the indicated treatments, as determined by crystal violet staining.

### 2.6. Oxidative Stress Determination

Intracellular ROS production was assessed using Dojindo’s Highly Sensitive DCFH-DA ROS Assay Kit according to the manufacturer’s instructions. This probe overcomes limitations of conventional DCFH-DA, including weak fluorescence intensity and high background signal, and the included loading buffer helps preserve cellular viability during the assay. Briefly, 2 × 10^4^ cells were seeded per well in black 96-well plates and allowed to attach overnight, then treated with the indicated concentrations of erastin for 6 h. Culture medium was removed, and the Highly Sensitive DCFH-DA working solution was added. Cells were incubated for 30 min, washed twice with HBSS, and fluorescence was measured using a Tecan Infinite 200 microplate spectrofluorometer (Tecan US, Inc, Morrisville, NC, USA)

### 2.7. Measurement of the LIP and Lipid Peroxidation

The LIP was determined using FerroOrange probe (Dojindo Laboratories, Rockville, MD, USA) according to the manufacturer’s instructions. Briefly, a total of 2 × 10^5^ cells were plated in 6-well plates and cultured for 24 h. Cells were then labeled with 1 μM FerroOrange in HBSS for 30 min at 37 °C. Samples were analyzed on a Becton Dickinson LSR II flow cytometer (BD Biosciences, Franklin Lakes, NJ, USA) at 561 nm (excitation) and 582 nm (emission). A total of 10,000 events were obtained per sample. The obtained data were analyzed using FlowJo v10.10.0 software (BD Biosciences, Ashland, OR, USA).

Lipid peroxidation was measured with Lipid Peroxidation Probe-BDP 581/591 C11 (Dojindo, Rockville, MD, USA), as we previously described [19]. Briefly, a total of 2 × 10^5^ cells were plated in 6-well plates and cultured for 24 h before exposure to the appropriate treatment conditions. After treatment, cells were labeled with 5 μM Lipid Peroxidation Probe in HBSS for 25 min at 37 °C and analyzed by flow cytometry on a Becton Dickinson LSR II flow cytometer using channels for Texas Red (reduced dye; 561 nm for excitation and 590 nm for emission) and fluorescein isothiocyanate FITC (oxidized dye; 488 nm for excitation and 530 nm for emission) simultaneously. A total of 10,000 events were obtained per sample. The obtained data were analyzed using FlowJo v10.10.0 software (FlowJo, LLC, Ashland, OR, USA).

### 2.8. Determination of Apoptosis

Apoptosis was assessed by flow cytometry using an Alexa Fluor 488 Annexin V/Dead Cell Apoptosis Kit (V13245, Invitrogen, Carlsbad, CA, USA), according to the manufacturer’s instructions, and analyzed by flow cytometry using a Becton Dickinson LSR II flow cytometer at 488 nm (excitation) and 530 nm and 575 nm (emission) (Becton Dickinson, Vernon Hills, IL, USA). Flow cytometry results were analyzed using FlowJo v10.10.0 Software.

### 2.9. Statistics

All data were evaluated using GraphPad Prism (https://www.graphpad.com/) (GraphPad Software, San Diego, CA, USA). Results are expressed as the mean ± SD, and *p* < 0.05 was considered significant. Statistical analyses were performed using one-way analysis of variance (ANOVA) followed by Tukey’s multiple comparison test or (un)paired Student *t*-test. Statistical significance was indicated with asterisks: * *p* < 0.05, ** *p* < 0.01, *** *p* < 0.001, and **** *p* < 0.0001.

## 3. Results

### 3.1. Generation and Characterization of ρ^0^ Cells and Transmitochondrial Cybrids

**Figure 1:**
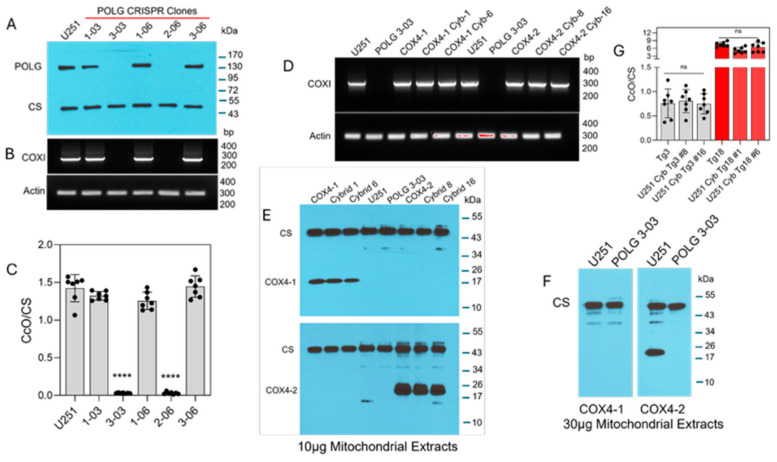
Characterization of POLG-KO clones and COX4 transmitochondrial cybrids. (**A**) Representative Western blot analysis of POLG protein expression in CRISPR/Cas9-generated POLG-KO clones, (*n* = 3). (**B**) Representative PCR analysis of COXI expression in POLG-KO clones; ACTIN served as the nuclear gene control (*n* = 2). (**C**) CcO enzymatic activity in POLG-KO clones compared with parental cells. Data are presented as the mean ± SD from three independent experiments. Statistical analysis was performed using one-way ANOVA followed by Tukey’s post hoc test (**** *p* < 0.0001). ns: not significant. (**D**) Representative PCR analysis of COXI expression in parental cells, mitochondrial donors, and cybrids; ACTIN served as a control, (*n* = 2). (**E**) Representative Western blot showing expression of COX4 isoforms in parental cells and COX4-1 and COX4-2 cybrids; 10 µg of mitochondrial extract was loaded per lane. CS served as the loading control, (*n* = 2). (**F**) Representative Western blot analysis of U251 parental and ρ^0^ cells (30 µg mitochondrial protein). COX4-2 expression is detectable in U251 parental cells but absent in ρ^0^ cells. CS served as the loading control, (*n* = 1). (**G**) CcO enzymatic activity in COX4-2- and COX4-1-expressing parental and cybrid cells. Data are presented as the mean ± SD from three independent experiments. Statistical analysis was performed using one-way ANOVA followed by Tukey’s post hoc test. ns: not significant.

ETC function requires the transcription of mtDNA, which is mediated by the catalytic subunit of mitochondrial DNA polymerase, POLG [30,31]. Therefore, to investigate the contribution of mitochondria in the regulation of ferroptosis in glioma cells, we used CRISPR gene editing to generate U251 POLG-KO cells. Loss of POLG expression was confirmed by Western blot in two clones (3-03 and 2-06), and mtDNA depletion was verified in these cells by PCR amplification of the mitochondrial COXI gene, which encodes subunit I of CcO (Figure 1A,B). Because COXI is a catalytic subunit of CcO, POLG depletion is also expected to abolish CcO activity. Indeed, both ρ^0^ clones exhibited complete loss of CcO activity (Figure 1C), consistent with the absence of COXI amplification. Together, these data demonstrate efficient CRISPR-mediated POLG disruption and the successful generation of mtDNA-depleted ρ^0^ U251 cells.

To determine whether mitochondria containing the COX4-1 isoform confer differential sensitivity to erastin, we generated transmitochondrial cybrids by fusing ρ^0^ U251 cells with enucleated cytoplasts from donor cells transgenically expressing either COX4-1 or COX4-2 (parental cells). COXI amplification was restored in COX4-1 cybrids (clones 1 and isoform-specific expression, with COX4-1 detected in cybrids 1 and 6 and COX4-2 detected in cybrids 8 and 16 (Figure 1E). Further concentration of the lysate was required to detect COX4-2 in the nuclear donor cells, indicating that the nuclear donor cells expressed COX4-2 at much lower levels than the transgenic parental cells did (30 µg per lane in Figure 1F versus 10 µg lysate per lane in Figure 1E). However, COX4 isoforms remained undetectable even in the highly concentrated POLG-KO lysates (Figure 1F). Following fusion, COX4-1- and COX4-2-expressing cybrids exhibited CcO activity at levels comparable to levels in the respective parental cells. In line with our previously published data [19,21], CcO activity was approximately tenfold lower in the COX4-2-expressing cybrids and parental cells than it was in the COX4-1-expressing counterparts (Figure 1G).

### 3.2. COX4-1-Expressing Cybrids Are Sensitive to Erastin but Exhibit Reduced Labile Iron, Cystine Uptake, and Expression of SLC7A11 and GPX4

**Figure 2:**
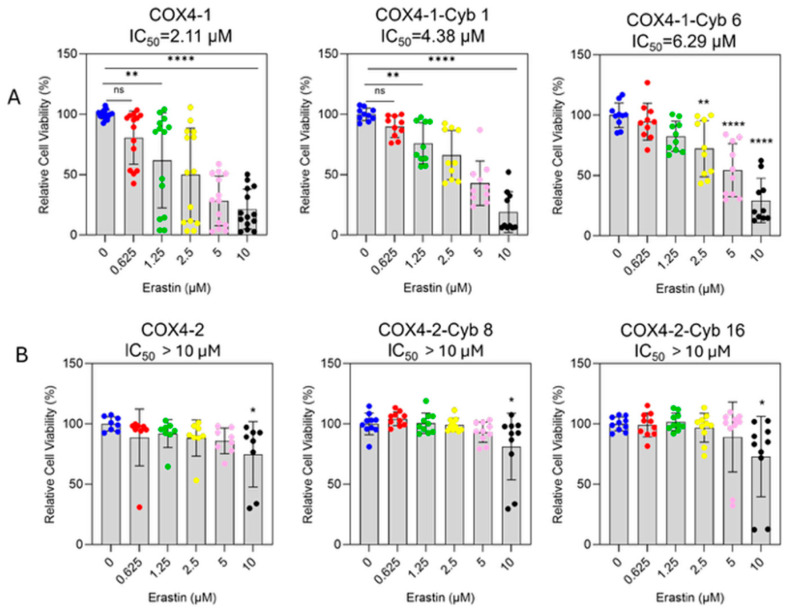
Differential sensitivity of COX4-1- and COX4-2-expressing cells to erastin treatment. (**A**) Dose–response curves showing the effect of erastin (0–10 µM) on cell viability in COX4-1-expressing parental and cybrid cells (clones 1 and 6). Cell viability was determined by crystal violet staining after 24 h of treatment. (**B**) Dose–response analysis of COX4-2-expressing parental and cybrid cells under the same conditions. Data are expressed as the mean ± SD from three independent experiments. Statistical significance was assessed using one-way ANOVA followed by Tukey’s post hoc test; significance is indicated as *p* < 0.05 (*), *p* < 0.01 (**) and *p* < 0.0001 (****). Cyb: cybrid.

To assess erastin sensitivity, cell viability was determined in parental mitochondrial donor cells and cybrid cells treated with increasing concentrations of erastin (0–10 μM, 24 h). Erastin inhibited the survival of COX4-1-expressing parental and cybrid cells in a dose-dependent manner (Figure 2A). The half-maximal inhibitory concentrations (IC_50_) were 2.11 μM for COX4-1-expressing parental cells and 4.48 μM and 6.29 μM for COX4-1-expressing cybrids 1 and 6, respectively. In contrast, erastin did not induce significant toxicity in COX4-2-expressing counterparts (Figure 2B).

**Figure 3:**
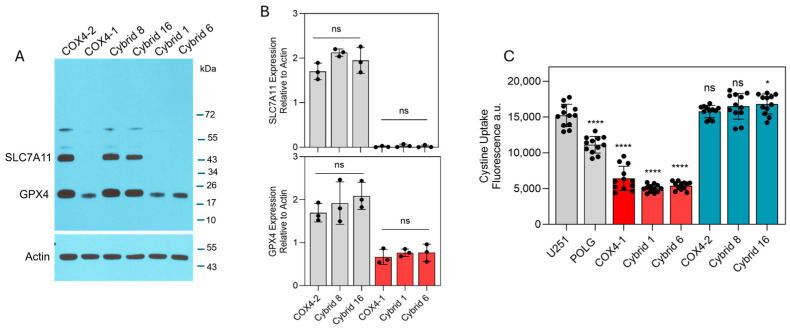
Expression of ferroptosis-related proteins and cystine uptake in COX4-1- and COX4-2-expressing cells. (**A**) Representative Western blot showing expression of SLC7A11 and GPX4 in COX4-1- and COX4-2-expressing parental cells and the respective cybrids. ACTIN was used as a loading control. (**B**) Densitometric analysis of WB bands for SLC7A11 and GPX4 normalized to β-actin. Data represent the mean ± SD (n = 3). (**C**) Quantification of cystine uptake in COX4-1- and COX4-2-expressing parental cells and the respective cybrids. Data are presented as the mean ± SD from three independent experiments. Statistical analysis was performed using one-way ANOVA followed by Tukey’s post hoc test; significance is indicated as *p* < 0.05 (*) and *p* < 0.0001 (****). ns: not significant.

To identify intracellular factors that could underlie the differential effects of erastin in COX4-1- and COX4-2-expressing cells, key ferroptosis-associated factors, including labile iron levels, cystine uptake, and expression of SLC7A11 and GPX4, were examined. Western blot analysis revealed high SLC7A11 expression in the COX4-2-expressing parental and cybrid cells, whereas SLC7A11 was undetectable in the COX4-1-expressing counterparts. Furthermore, GPX4 expression was markedly lower in COX4-1-expressing parental and cybrid cells than in COX4-2-expressing counterparts (Figure 3A). Consistent with these findings, cystine uptake was significantly greater in the COX4-2-expressing parental and cybrid cells than in the COX4-1-expressing parental and cybrid cells (Figure 3B).

**Figure 4:**
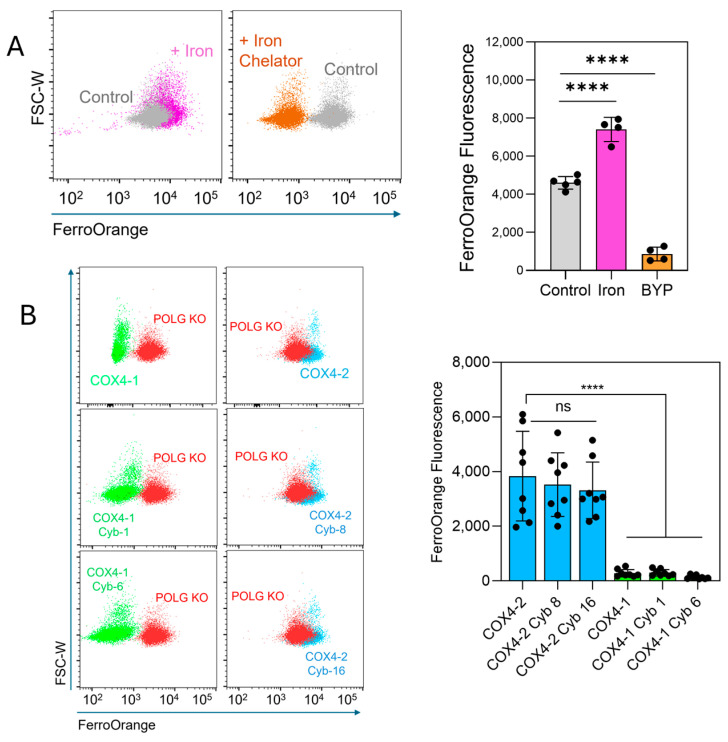
Assessment of intracellular labile iron in COX4-1- and COX4-2-expressing cells. (**A**) Representative flow cytometry scatter plots and quantification of FerroOrange fluorescence in U251 cells treated with iron (Fe^2+^, 100 µM, 30 min) or the iron chelator 2,2-bipyridyl (BYP, 100 µM), demonstrating probe specificity. Data are presented as the median ± SD from two independent experiments. (**B**) Flow cytometry scatter plots and quantification of FerroOrange fluorescence in POLG-KO cells and in COX4-1- and COX4-2-expressing parental and cybrid cells, showing differences in labile iron content among the indicated cell lines. Data are presented as the median ± SD from three independent experiments. Statistical analysis was performed using one-way ANOVA followed by Tukey’s post hoc test; significance is indicated as *p* < 0.0001 (****). Cyb: cybrid; ns: not significant.

The FerroOrange probe, which selectively binds intracellular ferrous iron (Fe^2+^), producing a fluorescence signal, was used to determine whether labile iron levels could explain the differential sensitivity to erastin. Treatment of U251 cells with ammonium iron(II) sulfate increased the fluorescence, whereas treatment with the iron chelator 2,2′-bipyridyl (BPY) reduced the signal (Figure 4A), confirming probe specificity. Quantitative analysis showed that labile iron levels were significantly lower in COX4-1-expressing parental cells (11.2-fold reduction), cybrid 1 cells (10.7-fold), and cybrid 6 cells (23.4-fold) than in COX4-2-expressing parental cells (Figure 4B). No significant differences were observed among COX4-2-expressing counterparts. Together, these results demonstrate that the mitochondria, rather than the nuclear background, controls the levels of ferroptosis-related factors and the response to erastin.

### 3.3. Erastin Promotes GSH Depletion and Oxidative Stress in COX4-1-Expressing Cybrids

**Figure 5:**
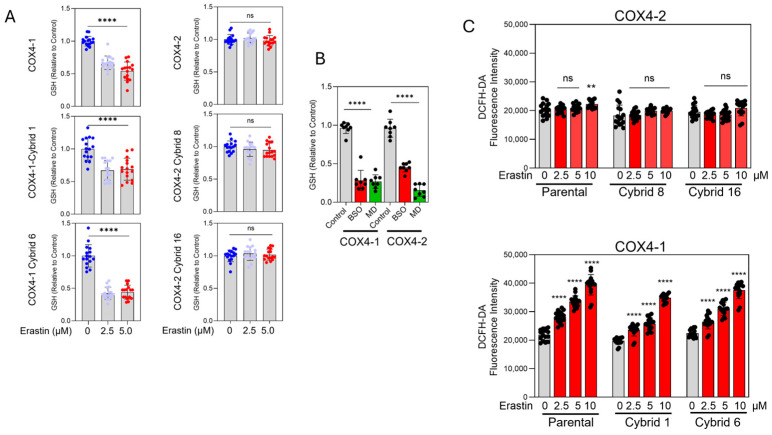
GSH content and oxidative stress in COX4-1- and COX4-2-expressing cells. (**A**) Quantification of GSH levels in COX4-1- and COX4-2-expressing and cybrid cells treated with erastin (0, 2.5, and 5 µM) for 24 h. (**B**) Quantification of GSH levels in COX4-1- and COX4-2-expressing cells following treatment with BSO or MD. (**C**) Quantification of oxidative stress in COX4-1- and COX4-2-expressing parental and cybrid cells, measured with the Highly Sensitive DCFH-DA ROS Assay Kit 6 h after treatment with erastin (0, 2.5, 5, and 10 µM). Data are presented as the mean ± SD from three independent experiments. Statistical significance was determined by one-way ANOVA with Tukey’s post hoc test; significance is indicated as *p* < 0.01 (**) and *p* < 0.0001 (****). ns: not significant.

Erastin reduced GSH content to approximately 38–40% of the control levels in COX4-1-expressing parental and cybrid cells. In contrast, GSH levels remained largely unchanged in COX4-2-expressing parental and cybrid cells after erastin exposure (Figure 5A), correlating with their relative resistance to the drug (Figure 2B). As expected, treatment with BSO or MD promoted GSH depletion in all COX4-1- and COX4-2-expressing cells, confirming the specific and differential effects of erastin on COX4-1- and COX4-2-expressing cells (Figure 5B).

Because GSH depletion facilitates lipid ROS accumulation, we next investigated whether erastin preferentially induces oxidative stress in COX4-1-expressing cells. Using the highly sensitive DCFH-DA probe, we measured intracellular ROS levels 6 h after treatment initiation, before cell death was evident. Erastin induced a dose-dependent increase in ROS in COX4-1-expressing parental and cybrid cells but not in their COX4-2-expressing counterparts (Figure 5C). These findings indicate that erastin selectively promotes GSH depletion and oxidative stress in COX4-1-expressing cells, revealing a mitochondrial composition–dependent redox vulnerability.

### 3.4. Erastin Induces Apoptosis, Not Ferroptosis, in COX4-1-Expressing Cybrids

**Figure 6:**
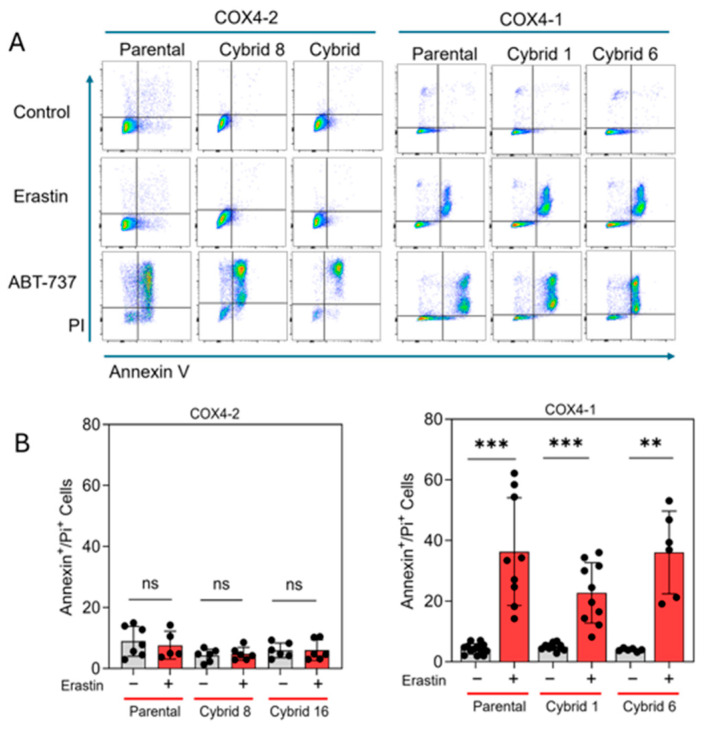
Apoptosis analysis in COX4-1- and COX4-2-expressing cells. (**A**) Representative flow cytometry scatter plots (FlowJo) of Annexin V/PI staining in COX4-1- and COX4-2-expressing parental and cybrid cells under control conditions or following treatment with erastin or ABT-737 as a positive control. (**B**) Quantification of Annexin V/PI-positive cells in control and erastin-treated samples. Data are presented as the median ± SD from three independent experiments. Statistical significance was assessed using one-way ANOVA with Tukey’s post hoc test; significance is indicated as *p* < 0.01 (**) and *p* < 0.001 (***). ns: not significant.

**Figure 7:**
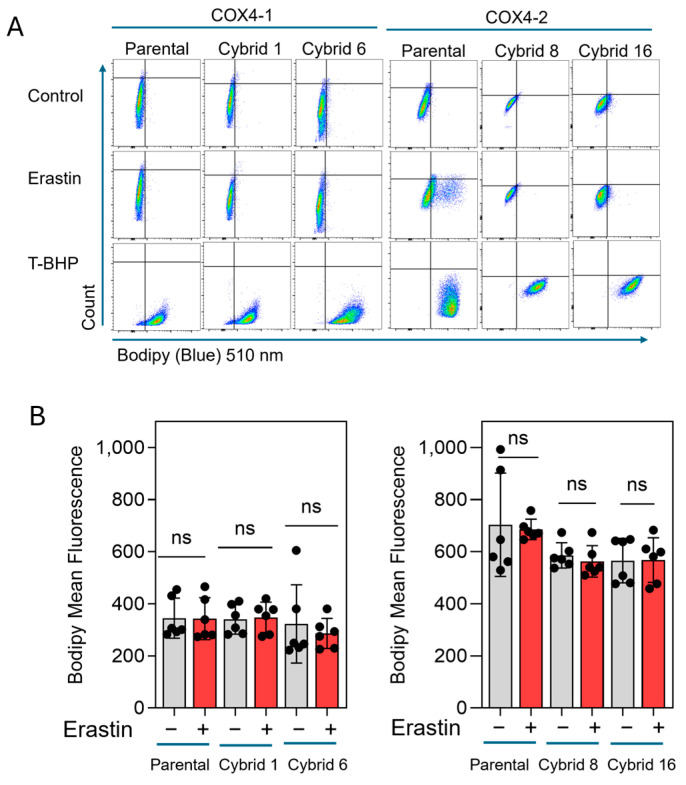
Lipid peroxidation in COX4-1 and COX4-2-expressing cells. (**A**) Representative flow cytometry scatter plots (FlowJo) of Lipid Peroxidation Probe-BDP 581/591 C11 staining in COX4-1- and COX4-2-expressing parental and cybrid cells under control conditions or following treatment with erastin or t-BHP (500 µM) for 2 h as a positive control. (**B**) Quantification of BODIPY fluorescence in control and erastin-treated samples. Data are presented as the median ± SD from three independent experiments. Statistical analysis was performed using one-way ANOVA with Tukey’s post hoc test; ns: not significant.

Our previous study indicated that erastin-induced cell death is not mediated by lipid peroxidation in radioresistant U251 cells, which exclusively express COX4-1 [19]. To further define the mode of cell death induced by erastin in the COX4-1-expressing cybrids, we assessed apoptosis in COX4-1- and COX4-2-expressing parental and cybrid cells after treatment with erastin, 5 µM for 24 h. In the COX4-1-expressing cells, the proportion of Annexin V/PI-positive cells increased dramatically from ~30% to 50%. In contrast, COX4-2-expressing cells showed no change in apoptosis under the same conditions. Treatment with ABT-737 (35 µM for 24 h), a known apoptosis inducer [32], confirmed that both cell lines and the respective cybrids were highly sensitive to apoptosis, indicating that the effect of erastin is specific to COX4-1-expressing cells (Figure 6). Consistent with our previous results, lipid peroxidation was nearly absent in both COX4-1- and COX4-2-expressing cells following erastin treatment (5 µM for 24 h), whereas all cells displayed lipid peroxidation in response to tertiary butyl hydroperoxide (t-BHP, 500 µM for 2 h) (Figure 7). These results suggest that erastin predominantly induces apoptotic rather than ferroptotic cell death in COX4-1-expressing cells.

## 4. Discussion

This study establishes a direct mechanistic link between mitochondrial composition and redox-dependent cell death pathways in GBM. Using CRISPR-mediated POLG-KO and transmitochondrial cybrid technology, we generated U251 ρ^0^ cells and reconstituted them with mitochondria containing either the COX4-1 or COX4-2 isoform of CcO. This approach allowed us to isolate mitochondrial contributions to erastin sensitivity independent of nuclear background effects. Restoration of mitochondrial DNA and respiratory function in cybrids faithfully reproduced the phenotypes of the respective mitochondrial donors, confirming that mitochondria are the primary determinants of redox balance and cell fate under ferroptotic stress.

Transmitochondrial cybrid models have been extensively used to dissect the functional contribution of mitochondria to cancer metabolism and therapy resistance. By placing distinct mitochondrial genotypes into a common nuclear background, this system provides a powerful means to define mitochondrial-dependent determinants of cellular phenotype. For instance, Ishikawa et al. [33] demonstrated that mitochondrial DNA mutations promoting enhanced ROS generation conferred increased metastatic potential in tumor cells, establishing a direct link between mitochondrial genotype and cancer aggressiveness. Similarly, Park et al. [34] showed that specific mitochondrial haplotypes altered oxidative phosphorylation and tumorigenic properties in breast cancer cybrids. In glioma, it has been demonstrated that mitochondrial governs CD133 expression, bioenergetic profiles, and temozolomide sensitivity [21,23]. More recent studies have extended the use of cybrids to explore how mitochondrial–nuclear crosstalk modulates resistance to apoptosis and ferroptosis [35,36]. Collectively, these studies underscore the versatility of transmitochondrial cybrid systems for elucidating the mitochondrial determinants of cancer cell behavior and therapeutic vulnerability.

Building on this foundation, our data reveal that the COX4 isoform context is a critical determinant of erastin sensitivity and redox-regulated cell death in glioma cells. Our findings reveal that COX4-1-expressing glioma cells and cybrids are markedly sensitive to erastin, undergoing apoptotic rather than ferroptotic death. In contrast, COX4-2-expressing cells and cybrids displayed resistance to erastin and showed features indicative of ferroptosis tolerance. This dichotomy suggests that mitochondrial composition—particularly COX4 isoform context—fundamentally influences redox metabolism, cystine utilization, and cell death susceptibility.

Erastin is classically understood to induce ferroptosis by inhibiting Xc^−^, leading to cystine depletion, GSH loss, and accumulation of lipid peroxides [1,2]. Surprisingly, COX4-1-expressing cells treated with erastin exhibited significant GSH depletion and oxidative stress but did not display typical ferroptotic markers such as lipid peroxidation or GPX4-dependent lethality, indicating a shift toward apoptotic mechanisms [19]. Similar noncanonical erastin responses have been described in certain cellular contexts in which GPX4 expression or cystine uptake is impaired [37,38,39]. The reduced expression of GPX4 and SLC7A11 observed in COX4-1-expressing cells may explain this apoptotic phenotype, as both genes are required to buffer oxidative stress and maintain redox homeostasis.

The low level of labile iron in COX4-1-expressing cells likely further limits ferroptotic initiation, since iron availability is essential for lipid peroxide propagation [10,40]. This reduction in unbound intracellular iron could stem from altered mitochondrial iron handling linked to COX4-1-dependent differences in respiratory chain activity and ROS signaling. Several studies have shown that mitochondrial dysfunction modulates cellular iron homeostasis and the expression of iron-regulatory genes [41,42,43,44,45]. In contrast, COX4-2-expressing cells had a larger LIP and higher cystine uptake, as well as preserved GSH pools, thereby conferring resistance to erastin-induced oxidative collapse.

These results align with emerging evidence that mitochondrial metabolic states dictate ferroptosis sensitivity. For example, Gaschler et al. [46] demonstrated that mitochondria-driven NADPH consumption controls ferroptosis execution, while Bersuker et al. and Doll et al. [47,48] reported that cells with higher mitochondrial activity exhibit altered lipid remodeling and antioxidant responses. Similarly, Gotorbe et al. [49] found that metabolic rewiring toward oxidative phosphorylation enhances ferroptosis resistance through improved redox buffering. Our study adds to this body of work by showing that specific mitochondrial subunit isoforms, such as COX4-1 versus COX4-2, can act as intrinsic determinants of redox vulnerability.

The differential regulation of GPX4 and SLC7A11 observed here also resonates with previous findings that stress-responsive transcription factors, including NRF2 and ATF4, control cystine transport and antioxidant gene networks [50,51]. Cells expressing the stress-responsive COX4-2 isoform may maintain higher NRF2 activity or enhanced mitochondrial-to-nuclear signaling, promoting adaptation to oxidative challenges. Conversely, COX4-1 cells appear metabolically less flexible, predisposing them to apoptotic death when cystine and GSH synthesis are compromised.

### Limitations of the Study

Several limitations should be acknowledged. Although transmitochondrial cybrids provide a robust system with which to dissect mitochondrial-dependent effects from nuclear influences, this study was conducted using a single nuclear genetic background. Thus, it remains to be determined whether similar COX4-isoform-dependent effects on redox metabolism and erastin sensitivity would occur across other glioma or non-glioma cell types. Furthermore, while cybrids recapitulate mitochondrial contributions in isolation, they cannot fully mimic the complexity of in vivo tumor microenvironments, where stromal and immune interactions, oxygen gradients, and nutrient availability shape redox homeostasis and cell death responses. Additional experiments using complementary cell lines or in vivo mouse models would strengthen the generalizability of these findings. Finally, although our data support apoptotic mechanisms in COX4-1 cells, future studies incorporating the direct assessment of mitochondrial iron pools and iron–sulfur cluster dynamics will be important to confirm the mechanistic link between mitochondrial identity, iron handling, and oxidative stress responses.

## 5. Conclusions

Collectively, our findings support a model in which the COX4 isoform identity governs the balance among mitochondrial respiration, iron handling, and redox regulation, ultimately determining whether glioma cells undergo apoptosis or ferroptosis in response to erastin. COX4-1-containing mitochondria restore cytochrome c oxidase activity and reprogram iron and redox metabolism to suppress erastin-induced ferroptosis and promote apoptotic cell death, whereas COX4-2-containing mitochondria lack this capacity and remain insensitive to erastin. This framework highlights a broader role for mitochondrial diversity in shaping therapeutic responses in glioma and potentially other cancers.

## Data Availability

The original contributions presented in this study are included in the article material. Further inquiries can be directed to the corresponding author.
